# The mechanism of a one-substrate transketolase reaction

**DOI:** 10.1042/BSR20180246

**Published:** 2020-08-04

**Authors:** Olga N. Solovjeva, Marina V. Kovina, Maria G. Zavialova, Victor G. Zgoda, Dmitrii S. Shcherbinin, German A. Kochetov

**Affiliations:** 1 Belozersky Institute of Physico-Chemical Biology, Lomonosov Moscow State University, 119992 Moscow, Russian Federation; 2 Advanced Cell Technologies Department, Sechenov First Moscow State Medical University (Sechenov University), Trubetskaya Street 8, 119991 Moscow, Russian Federation; 3 Institute of Biomedical Chemistry, Pogodinskaya 10, 119121 Moscow, Russian Federation

**Keywords:** mass-spectrometry, thiamine catalysis, transketolase

## Abstract

Transketolase catalyzes the transfer of a glycolaldehyde residue from ketose (the donor substrate) to aldose (the acceptor substrate). In the absence of aldose, transketolase catalyzes a one-substrate reaction that involves only ketose. The mechanism of this reaction is unknown. Here, we show that hydroxypyruvate serves as a substrate for the one-substrate reaction and, as well as with the xylulose-5-phosphate, the reaction product is erythrulose rather than glycolaldehyde. The amount of erythrulose released into the medium is equimolar to a double amount of the transformed substrate. This could only be the case if the glycol aldehyde formed by conversion of the first ketose molecule (the product of the first half reaction) remains bound to the enzyme, waiting for condensation with the second molecule of glycol aldehyde. Using mass spectrometry of catalytic intermediates and their subsequent fragmentation, we show here that interaction of the holotransketolase with hydroxypyruvate results in the equiprobable binding of the active glycolaldehyde to the thiazole ring of thiamine diphosphate and to the amino group of its aminopyrimidine ring. We also show that these two loci can accommodate simultaneously two glycolaldehyde molecules. It explains well their condensation without release into the medium, which we have shown earlier.

## Introduction

Transketolase (TK; EC 2.2.1.1), a typical representative of the thiamine diphosphate-dependent enzymes, requires for its activity thiamine diphosphate as a coenzyme as well as divalent cations: Mg^2+^, Mn^2+^, or Ca^2+^ that ensure binding between the coenzyme and the apoprotein [1,2]. TK is widespread in nature and has been found in all studied organs and tissues of animals, plants and also in microorganisms [3–5].

TK is involved in the production of precursors of many vitally important substances in the living cell, nucleotides, coenzymes, aromatic amino acids, and others [6].

Together with transaldolase, TK is involved in the interaction between glycolysis and the pentose phosphate pathway of carbohydrate metabolism [7], which enables the cell to become adapted to different metabolic requirements.

Transketolase catalyzes the reversible cleavage of the C2-C3 bond in keto sugars (donor substrates RCHOH**COCH_2_OH**) [3,4,8]. The cleaved-off two-carbon fragment of the keto substrate (the active glycolaldehyde) becomes covalently bound to ThDP, namely (as is currently believed) to its thiazole ring and then, without being released into the medium, is transferred to the aldo sugar (the acceptor substrate R_1_CHO). The rest of the donor substrate is released into the medium as the product aldo sugar RCHO [9].

$$\text{RCHOH}\mathrm{COC}H_{2}\mathrm{OH}+{\text{ R}}_{1}\text{CHO }=\text{ RCHO }+{\text{ R}}_{1}\text{CHOH}\mathrm{COC}H_{2}\mathrm{OH}$$

In the absence of an aldo sugar a one-substrate reaction takes place – only the donor substrate is utilized [10,11].

$$2\text{ RСHОН}\mathrm{COC}H_{2}\mathrm{OH}=\;2\text{ RCHO }+\text{ Erythrulose}$$

**Scheme 1:**
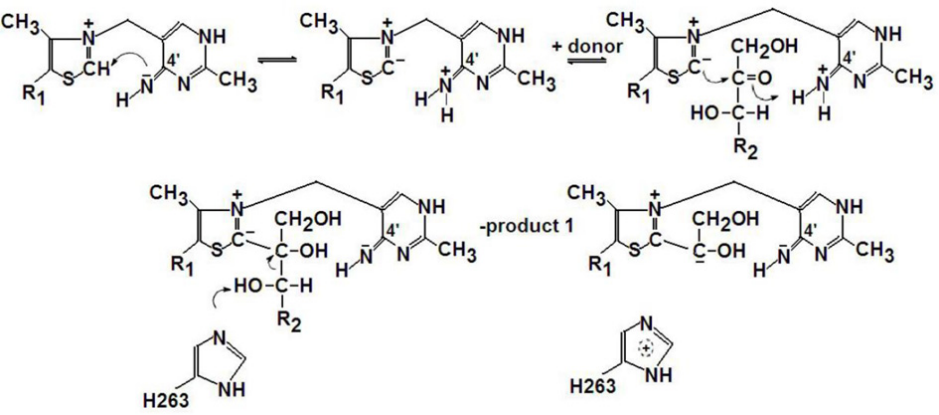
The classical ThDP activation mechanism (According to Shnider and Lindguist 1998 [9]).

The mechanism of this reaction remained unknown, here we aim to understand it. The first half of this reaction is predictable, as we know that the ThDP activation mechanism includes two closely linked processes [9]: deprotonation of the C2 thiazole ring (the site of the subsequent keto substrate binding) and stabilization of the resulting carbanion by the aminopyridine moiety (Scheme 1).

As follows from Scheme 1, the classical mechanism of the two-substrate transketolase reaction assumes the binding of the first substrate (ketose) and the glycol aldehyde residue (GliA) which is cleaved from it, to the C2 of the thiazole ring. Therefore, it is presently accepted that the second substrate (aldose) becomes bound to the GliA residue rather than to C2. However, in the one-substrate reaction the second substrate is also ketose, and another GliA residue is cleaved off from the second ketose molecule and condenses with the first one [10,11]. It seems logical to suppose therefore that the second ketose molecule, like the first one, might be bound to and cleaved at the C2 of thiazole, whereas the first GliA residue moves over to an alternative binding site. If an alternative binding site exists, a few options are possible *a priori*:

1. The first GliA moiety is bound to the thiazole ring of ThDP, and the second substrate molecule binds to the pre-attached GliA (this option coincides with the presently accepted mechanism of the two-substrate transketolase reaction).
2. The first GliA moiety is bound to the thiazole ring, while the second substrate molecule binds to another site, for example, to the aminopyrimidine ring or to the protein.
3. The first GliA moiety binds to (is transferred to) another binding site, for example, aminopyrimidine ring via a Schiff base, after which the second keto substrate molecule binds to the thiazole ring and is cleaved producing the second GliA molecule. Subsequently, the two GliA molecules undergo condensation forming erythrulose at the initial or another binding site.

In the present study we employed mass-spectrometry and 3D modeling to determine which of the above-mentioned options is correct.

### Experimental

The following chemicals were used: α-glyceraldehyde 3-phosphate dehydrogenase from rabbit muscle, NAD^+^, ThDP, glycylglycine, CaCl_2_, formic acid (Sigma), dithiothreitol, sodium arsenate (Fluka, Germany), Sephadex G-50 (Pharmacia, Sweden). Other reactants were of extra pure grade.

### Transketolase purification

Transketolase was isolated from baker’s yeast *Saccharomyces cerevisiae* as described earlier [12], aliquoted and stored frozen in 20 mM potassium phosphate buffer. The enzyme was homogeneous by SDS-PAGE and exhibited a specific activity of 30 U/mg. The concentration of TK was determined spectrophotometrically using A^1%^_1cm_ = 14.5 at 280 nm [13]. Prior to use, the TK solution was passed through a Sephadex G-50 column, equilibrated with 50 mM glycylglycine, pH 7.6.

### Preparation of holotransketolase

TK at 10–15 mg/ml was supplemented with 2.5 mM CaCl_2_ and ThDP, whose concentration was twice the molar concentration of TK (i.e. one ThDP for each active center).

### Preparation of the phosphopentose mixture

The barium salt of the phosphopentose mixture containing xylulose 5-phosphate and ribose 5-phosphate was prepared using an earlier described method [14]. The barium salt was converted to the potassium salt before use.

### Measurement of transketolase activity

The catalytic activity of TK was measured using spectrophotometry by the rate of NAD^+^ reduction in a coupled system with glyceraldehyde 3-phosphate dehydrogenase [15]. The reaction mixture in the final volume of 1 ml contained 50 mM glycylglycine, 2.5 mM CaCl_2_, 1 mM sodium arsenate, 3.2 mM dithiothreitol, 0.1 mM ThDP, 1.6 mM NAD^+^, 3 units of glyceraldehyde 3-phosphate dehydrogenase, and 3.2 mg/ml potassium salt of a phosphopentose mixture, which was used as the substrate, at pH 7.6. The reaction was initiated by the addition of the phosphopentose solution.

### Determination of thiamine diphosphate

The concentration of ThDP was determined spectrophotometrically by measuring the optical density at 272.5 nm (using the molar extinction coefficient 7500 M^–1^cm^–1^) [16].

### Erythrulose determination

Erythrulose accumulation was performed in the following reaction mixture: 50 mM glycylglycine, 10 mg/ml TK, 0.1 mM CaCl_2_, 0.17 mM ThDP, 1 mM hydroxypyruvate (HPA), pH 7.6. After the total depletion of HPA in the one-substrate reaction (about 4 h), the reaction mixture was treated with 10% chloric acid, spinned off from the protein precipitate, and neutralized with KOH. Erythrulose was determined by the method described earlier [17].

### ESI-MS and MS/MS analysis

The samples were in 40 mM ammonia, 40 mM formic acid and 5% TCA, pH 4.0, dissolved in water.

We analyzed samples using an LTQ Orbitrap mass spectrometer. A sample was injected at 3 μl/min with nebulizer gas flow of 8 l/min. The temperature of the inlet capillary was 270°C, and the capillary voltage, 3.6 kV. The mass spectra of positive ions were measured using a FTMS analyzer with mass resolution of 60,000 in the mass range of 200–1000 Da. The mass accuracy was above 5 ppm. We used tandem mass-spectra to clarify the composition and structure of the analyte. The appropriate ions were isolated in a 2 Da mass window and subjected to collision-induced dissociation (CID). The resulting spectra were obtained by averaging 300 scans.

### Molecular 3D modeling

The spatial structures of TK with covalently bound GliA to ThDP were obtained from the Protein Data Bank (PDB) (PDBid 1GPU) [18]. Preliminary optimization of the structures was performed using the SYBYL 8.1 software package, which was also used for the additional modification of ThDP in which the second GliA was bound to the pyrimidine ring. The structures were minimized in vacuum using the Tripos force field and Gasteiger–Huckel atomic partial charges.

We have designed a preliminary model of the cofactor with two GliA residues bound to TK by adding a second GliA to the cofactor with covalently bound GliA (PDB id 1GPU), then we optimized the model by minimization and molecular dynamics.

Molecular dynamics simulations were performed using the Amber8 software package. Parameterization was carried out using the ff99-SB force field for protein molecules and the GAFF force field for cofactor atoms.

The TIP3P water model was used for solvation and Na+ ions were added for system neutralization.

At the first stage sequential minimization of the system was performed in vacuum and in a solvent (for 25,000 steps). The next stage included sequential heating of the system (up to 300 K) and pressure increase (up to 1 atm) during 40 ps using NVT and NPT ensembles. Particle mesh Ewald (PME) was employed to treat the long-range electrostatic interactions and the cut-off for the non-bonded interactions was set to 8 Å. The temperature was maintained using Langevin dynamics with a friction coefficient of 2 ps^-1^, and pressure was controlled with a Berendsen barostat. Molecular dynamics was performed on 10 ns trajectories with 2 fs time steps.

MD results were analyzed using the AMBER program tool ptraj and the VMD software [19].

## Results

### Identification and quantization of the one-substrate reaction product

In this study, we employed HPA as the substrate of the one-substrate transketolase reaction. Its product erythrulose was determined as equimolar to the initial HPA load divided by a factor two (Table 1). Therefore, this reaction is similar to that with xylulose-5-phosphate [10,11]. The reaction with HPA is irreversible since one of its products is CO_2_, and for this reason we expect that a greater proportion of ThDP should be bound to the reaction’s intermediate than in the reversible reaction with xylulose-5-phosphate.

**Table 1:** Consumption of HPA and formation of erythrulose in a one-substrate reaction catalyzed by TK

Initial HPA load, mM	Amount of erythrulose formed		
	Theoretically expected, mM	Actually formed, mM	% of the expected
1.0	0.5	0.48	96
0.5	0.25	0.23	92

Erythrulose was determined according to [17] after the total depletion of HPA and subsequent protein removal.

### Preparation of samples for mass-spectrometry

The TK holoenzyme (holoTK) was incubated with HPA for 4 h at room temperature in the presence or absence of NaCNBH_3_.

Following incubation the sample was passed through a Sephadex G-50 column equilibrated with 30 mM NH_4_OH and 40 mM formic acid, pH 6.5. The fractions containing protein with bound ThDP and ThDP derivatives (the intermediates) were collected. The protein was then denatured with 5% TCA and removed by centrifugation. The supernatant was used for mass spectrometry.

### Mass spectrometry of intermediates formed in the one-substrate transketolase reaction

**Figure 1:**
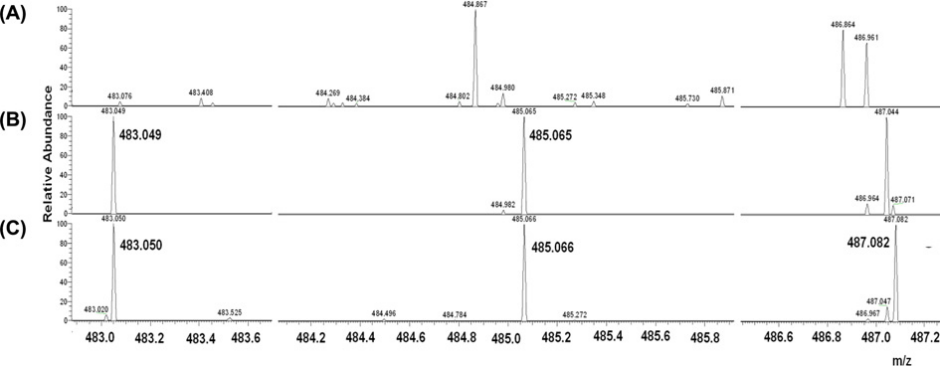
ESI-MS spectra of holoTK reaction medium in the range from 482.9 to 487.3 Da (**A**) ESI-MS spectra of holoTK in the absence of HPA and NaCNBH_3_; (**B**) in the presence of HPA; (**C**) in the presence of HPA and NaCNBH_3_.

Figure 1 shows mass spectra of ThDP intermediates formed in the first half of the one-substrate reaction, the binding and cleavage of the first HPA molecule as a substrate, in the absence and presence of NaCNBH_3_ (spectra 2 and 3, respectively) together with the control mass spectrum of the holoTK (spectrum 1).

Mass spectra in the wider range (from 480 to 550 Da) are given in Supplemental Figure S1.

Both in the presence and absence of NaCNBH_3_, the mass spectra of the first semi-reaction intermediates (spectra 3 and 2, Figure 1) reveal the appearance of 1^+^ charged molecular ions with *m/z* (mass) of 483.050 and 485.066, corresponding to the calculated sums of masses of GliA and ThDP (dehydroThDP): 60.0211 + 425.0449 (423.0292)= 485.0660 (483.0503).

The mass 487.082 appears only in the spectra of intermediates produced in the presence of NaCNBH_3_ (Figure 1, spectrum 3) and corresponds to the dihydride of the compound with mass 485: 485.066+2*(1.0078) = 487.082.

None of the expected masses, 483.050, 485.066, 487.082, appears on trace1 of Figure 1, the mass spectrum of holoTK, while it has a few contaminant peaks (484.867, 486.961). The approach to refine spectra from contaminant peaks is discussed in the Supplementary Material.

Let us now consider what information on the structure of these intermediates can be provided by their fragmentation.

### Fragmentation of intermediates of the one-substrate transketolase reaction

**Figure 2:**
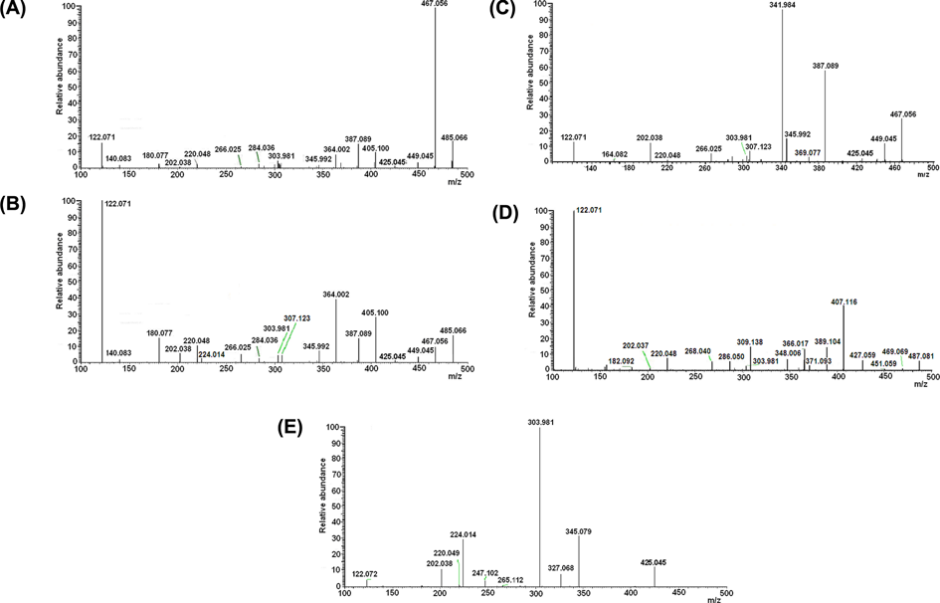
Typical ESI-MS/MS spectra of isolated intermediates of holoTK reaction with HPA (**A** and **B**): mass 485.066 Da; (**C**): mass 467.056 Da; (**D**): mass 487.081 Da; (**E**): mass 425.045 Da. Mass 425.045 was obtained from ESI-MS spectra of the TK holoenzyme in the absence of HPA and NaCNBH_3_.

The main fragment of intermediate 485.066 subjected to mild fragmentation is its dehydratation product with the experimentally obtained mass of 467.056, in a good agreement with the calculated value: 485.066 - 2*(1.0078) – 15.995 = 467.0557 (Figure 2A).

**Figure 3:**
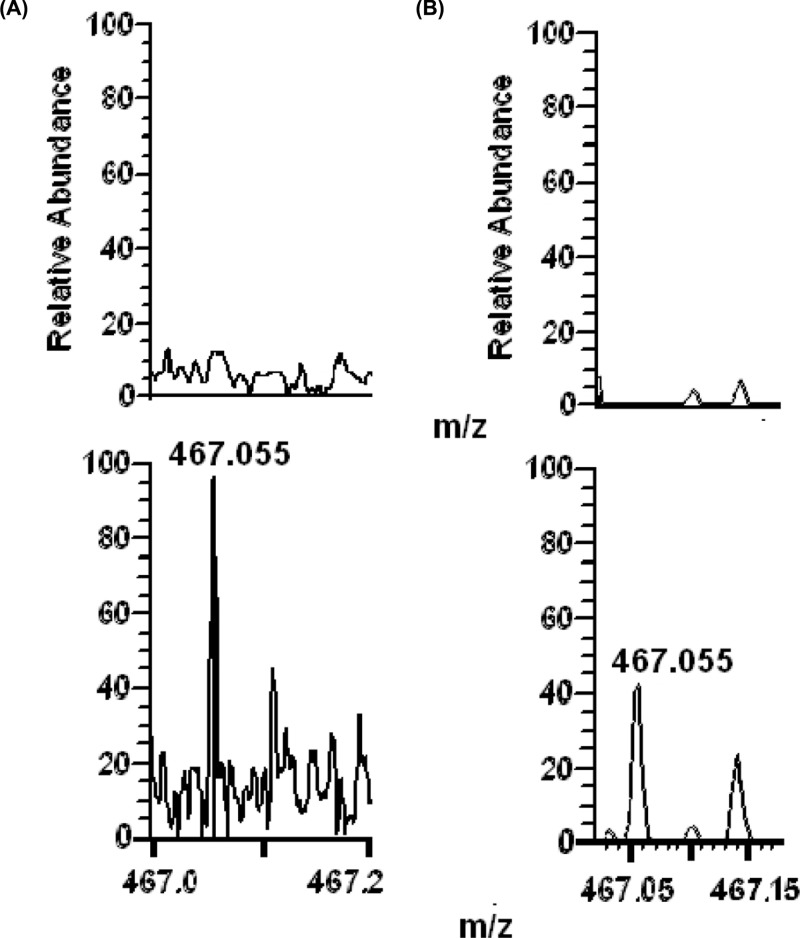
ESI-MS spectra of holoTK reaction medium in the range from 467 to 467.2 Da The mixtures of intermediates are produced by holoTK with HPA (lower graphs) or without HPA (upper graphs) in the absence (**A**) or presence (**B**) of NaCNBH_3_.

Under more rigid fragmentation conditions, the amplitude of mass 467.056 decreases while amplitudes of smaller masses increase, including mass 180.076, with mass values remaining unchanged (compare Figure 2B and Figure 2A). The peak 467.056 is also present in the main mass spectra (Figure 3A,B).

Fragments of mass 467.056 of the main mass spectrum are present in the fragmentation set of mass 485.066 (compare Figure 2C and Figure 2B), with exception of masses corresponding to dehydratation or deoxygenation (decreased by 18.011 or 15.994):

449.045 = 467.056 - 18.011
387.089 = 405.100 - 18.011
345.992 = 364.002 - 18.010
164.082 = 180.076 - 15.994
122.071 = 140.081 - 18.010

Thus, the peak 467.056 of the main spectrum is formed by the dehydrated intermediate 485.066, whose dehydratation may occur under non-fragmenting conditions either spontaneously or enzymatically.

**Scheme 2:**
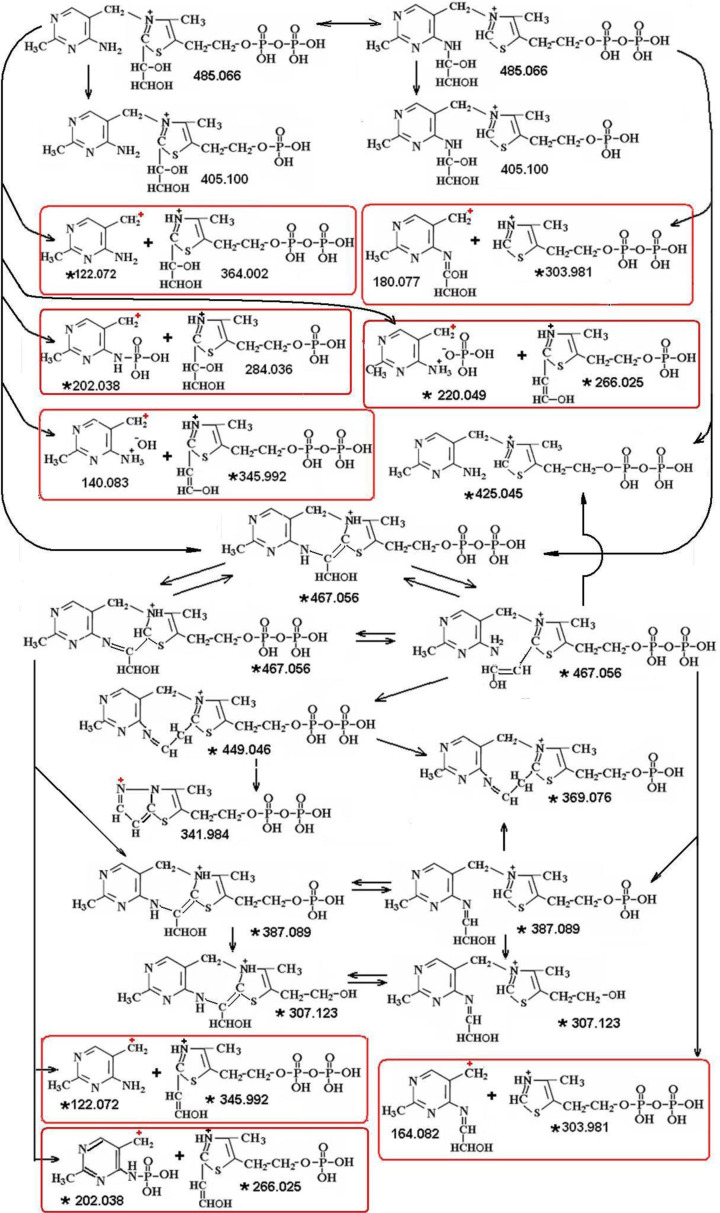
Proposed structures of the intermediate 485.066 Da and its fragments The lower part of the scheme shows the family of its main fragment 467.056 Da, which is also present as an independent intermediate in the TK-reaction medium.The framework combines complementary fragments. Repeating masses are marked with an asterisk (*), all fragments are charged positively (separation at the anode). The positive charge produced when the electron is separated from the radical upon breaking the bond is marked **+**.

Based on the masses we have determined the atomic composition (Table 2) and the sequence of fragmentation of intermediates 485.066 and 467.056 (Scheme 2). It includes:

- cleavage off of water (485.066 - 18.011 = 467.055, 467.056 - 18.011 = 449.045), phosphate (485.066 - 79.966 = 405.100), phosphate and water (485.066 - 18.011 - 79.966 = 387.089), diphosphate and water (485.066 - 18.011 - 159.933 = 307.122),
- dissociation of the GliA moiety with formation of the initial ThDP (425.045),
- breakage of the molecule between the rings with or without loss of phosphate or water: 122.072 + 345.992 (+18), 122 + 364, 122.072 + 284.036 (+80), 122.072 + 266.025 (+18+80), 220.049 + 266.025, 202.038 + 284.036 (266+18), ***180.077 + 303.981. 164.082+303.981.***

**Table 2:** MS/MS fragments of masses 425.045, 485.066, 487.082, and 467.056

Molecular formula	Calculated masses	Experimental fragments of the mass (*m/z*)			
		425	485	487	467
C_14_O_9_H_23_S_1_N_4_P_2_*	485.066	-	485.066	-	-
C_14_O_9_H_25_S_1_N_4_P_2_*	487.082	-	-	487.081	-
C_14_O_8_H_21_S_1_N_4_P_2_*	467.056	-	467.056	467.055	467.055
C_14_O_8_H_23_S_1_N_4_P_2_*	469.071	-	-	469.069	-
C_14_O_7_H_19_S_1_N_4_P_2_*	449.045	-	449.045	-	449.043
C_14_O_7_H_21_S_1_N_4_P_2_*	451.061	-	-	451.059	-
C_12_O_7_H_19_S_1_N_4_P_2_	425.045	425.045	425.045	-	425.044
C_12_O_7_H_21_S_1_N_4_P*_2_	427.061	-	-	427.059	-
C_14_O_6_H_22_S_1_N_4_P_1_*	405.100	-	405.100	-	-
C_14_O_6_H_24_S_1_N_4_P_1_*	407.116	-	-	407.116	-
C_14_O_5_H_20_S_1_N_4_P_1_*	387.089	-	387.089	-	387.089
C_14_O_5_H_22_S_1_N_4_P_1_*	389.105	-	-	389.105	-
C_8_O_9_H_16_S_1_N_1_P_2_*	364.002	-	364.002	-	-
C_8_O_9_H_18_S_1_N_1_P_2_*	366.018	-	-	366.017	-
C_12_O_4_H_18_S_1_N_4_P_1_	345.079	345.079	-	-	-
C_8_O_8_H_14_S_1_N_1_P_2_*	345.992	-	345.992	-	345.991
C_8_O_8_H_16_S_1_N_1_P_2_*	348.006	-	-	348.006	-
C_12_O_3_H_16_S_1_N_4_P_1_	327.068	327.068	-	-	-
C_8_O_7_H_12_S_1_N_2_P_2_*	341.984	-	-	-	341.983
C_14_O_2_H_19_S_1_N_4_ *	307.123	-	307.123	-	307.122
C_14_O_2_H_21_S_1_N_4_ *	309.138	-	-		-
C_6_O_7_H_12_S_1_N_1_P_2_	303.981	303.981	303.981	303.981	303.980
C_8_O_6_H_15_S_1_N_1_P_1_*	284.036	-	284.0355	284.036	-
C_8_O_6_H_17_S_1_N_1_P_1_*	286.052	-	-	286.050	-
C_8_O_5_H_13_S_1_N_1_P_1_*	266.025	-	266.024	-	266.025
C_8_O_5_H_15_S_1_N_1_P_1_*	268.040	-	-	268.040	
C_6_O_4_H_11_S_1_N_1_P_1_	224.014	224.014	224.014	224.014	-
C_6_O_4_H_13_S_1_N_1_P_1_	-	-	-	226.029	-
C_6_O_4_H_11_N_3_P_1_	220.049	220.049	220.048	220.048	220.048
C_6_O_3_H_9_N_3_P_1_	202.038	202.038	202.038	202.037	202.038
C_8_O_2_H_10_N_3_*	180.077	-	**180.077**	**180.076**	**-**
C_8_O_2_H_10_N_3_*	182.093	-	**-**	**182.092**	**-**
C_8_O_1_H_10_N_3_*	164.082	-	**-**	**-**	**164.082**
C_6_H_8_N_3_	122.072	122.072	122.071	122.071	122.072

The GliA-containing masses are marked with an asterisk (*).

Noteworthy are the two latter combinations given in bold: the masses 180.076 and 164.082 correspond to those of the pyrimidine ring of ThDP with bound GliA or its deoxo-derivative (Scheme 2).

**Scheme 3:**
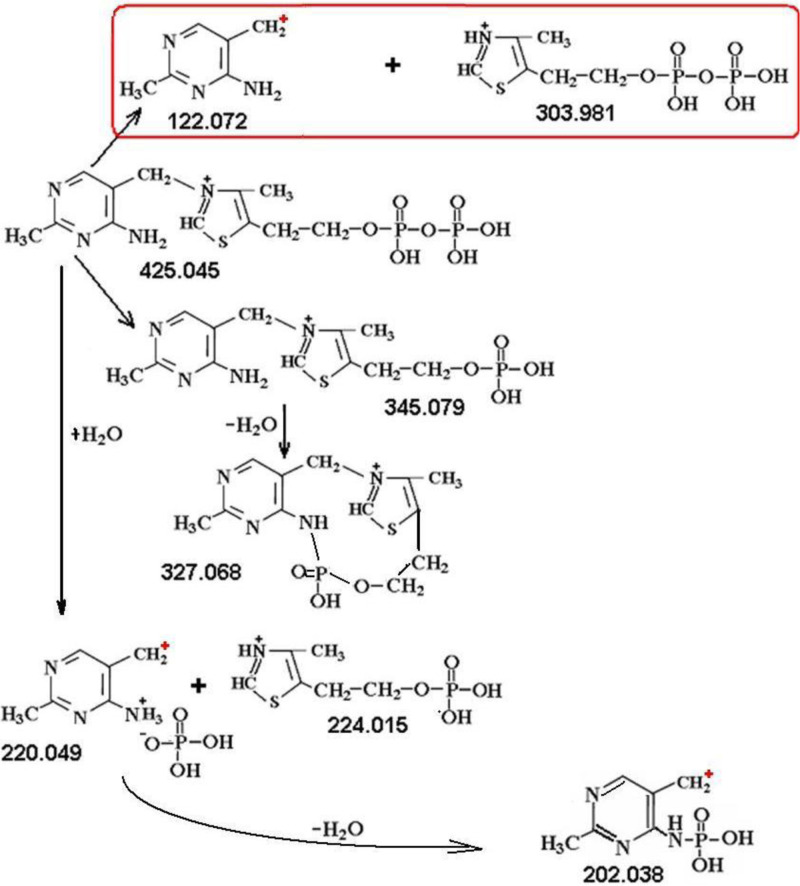
Structural formulas of proposed ThDP fragments The framework encloses complementary fragments. All fragments are charged positively (separation at the anode). The positive charge produced when the electron is separated from the radical upon breaking the bond is marked +.

To confirm the proposed structures, we fragmented ThDP as a compound with known structure (Figure 2E and Scheme 3). Majority of its fragmentation masses coincided with GliA-free fragment masses of intermediates: dephospho\dehydrates 345.079, 327.068, 265.112, 247.102 and mono-rings 122.072, 303.981, 224.015, 220.049, 202.038, see Table 2.

In the presence of NaCNBH_3_, the intermediate 485.066 is hydrogenated to mass 487.082. A comparison of the fragmentation spectra of intermediates 485.066 and 487.082 (Figure 2B,D, Table 2) suggests that hydrogenation does not prevent fragmentation of intermediate 487.082 at the same loci as for intermediate 485.066, but some of the masses, including the important mass 180.077, are increased by ∼2.015 Da, i.e. by two hydrogen masses: 180.077 + 2 x 1.0078=182.093.

## Discussion

A comparison of the fragmentation spectrum of ThDP as a substance of known structure with that of the intermediates 485, 467, and 487 whose structure we determined by mass spectrometry shows that the fragments identified by us as GliA-containing (marked with an asterisk in Table 2) were present only in the spectra of intermediates and completely absent in the fragmentation spectrum of ThDP, while most GliA-free peaks were observed both in the fragmentation spectra of intermediates and ThDP. Thus, the reliability of determining the structure of fragments by the mass spectrometry method was confirmed not only by the exact agreement between the expected and experimental masses (accurate to the third decimal place) but also by the expected presence/absence of GliA-containing/not-containing masses in the spectra of fragmentations of the intermediates and of the ThDP control.

The existence of covalent bonding of GliA with the amino-group of the pyrimidine ring is confirmed by the formation of mass 180.076 upon fragmentation of intermediate 485.050, as well as by the inverse correlation of its amplitude with that of the largest 467-fragment (compare A and B in Figure 2). The mass-identification of this product indicates that it corresponds to the structure of the GliA-adduct of the aminopyrimidine ring of ThDP.

It is interesting to note that the formation of mass 180.076 from the initial mass 485.065 is accompanied by dehydrogenation, so that the total mass of both fragments, 180.076+303.981 (-1.008) =483.050, corresponds to a spontaneously dehydrogenated product 483 of the native protein-bound intermediate 484^-^, shown in the Scheme 1. This is indicative of a possible spontaneous inter-conversion between forms 485 and 483, found in the protein-free mass spectra and confirms our assumption about the equally probable and reversible transformation of protein-bound carbanion 484^-^ into its protein-free derivatives 483 and 485.

**Scheme 4:**
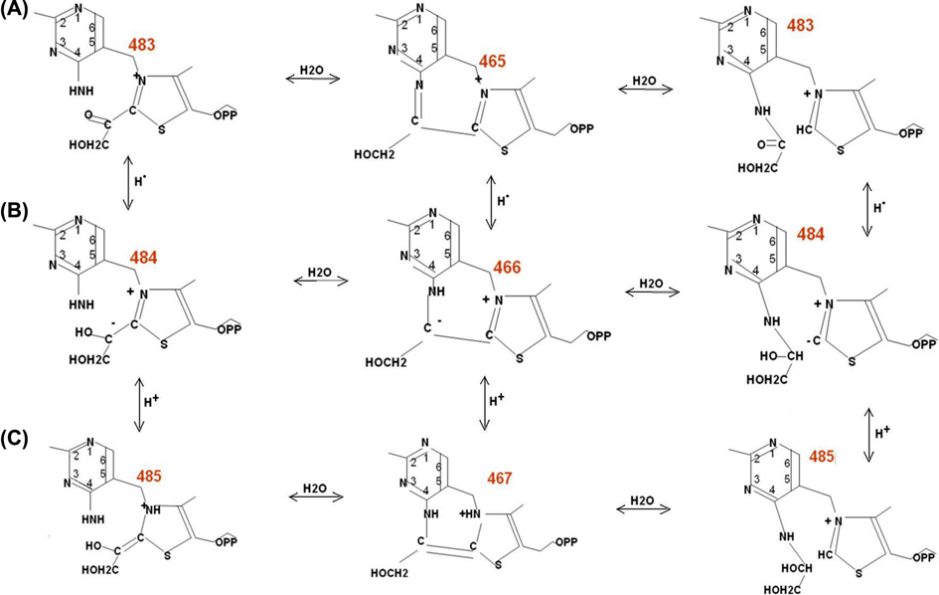
A tricycle as a structure enabling GliA exchange between the ThDP loci GliA transition is accompanied with (de)protonation and (de)hydrogenation of the intermediate.

The result obtained in the present study, the covalent binding of glycolaldehyde to the aminopyrimidine ring of ThDP, suggests that the amino group and the C2 of the thiazole ring are equally capable of binding the transferred residue of the ketosubstrate, the GliA moiety. Apparently, a binding equilibrium of GliA to these two loci of ThDP is possible through formation of a tricycle accompanied by reversible dissociation of water (Scheme 4).

The existence in the initial mass-spectra of intermediates 483, 485, and the dehydrated 467 offers a good support for the above Scheme. The intermediates 465, 466^-^, 467 should have tricyclic structures, since non-cyclic structures of this type are chemically unstable. The possibility of a tricycle formation was shown earlier for both ThDP [20] and its derivatives with aldehydes [21].

The observation of the fragment with mass 164.082 in the fragmentation spectrum of the dehydrated product 467 offers an additional proof for the existence of a covalent bond between GliA and the amino group of ThDP. The mass value (164.082) equals that of aminopyrimidine with deoxo-GliA (=180.076 – 15.994), the result of a loss of water during cyclization of intermediate 485 into 467.

Besides, a small peak 182.092, corresponding to a dihydride of adduct of aminopyrimidine with GliA (=180.076 + 2*1.0078), is seen in the fragmentation spectrum of mass 487, a cyanborohydride-reduced derivative of intermediate 485 (Figure 2D).

Therefore, we have found a second binding site for GliA, in addition to thiazolic C2: the aminogroup of pyrimidine. This rules out option 1 (introduction) as a possibility for overall catalysis, namely that the second substrate molecule binds to the first pre-attached GliA.

**Figure 4:**
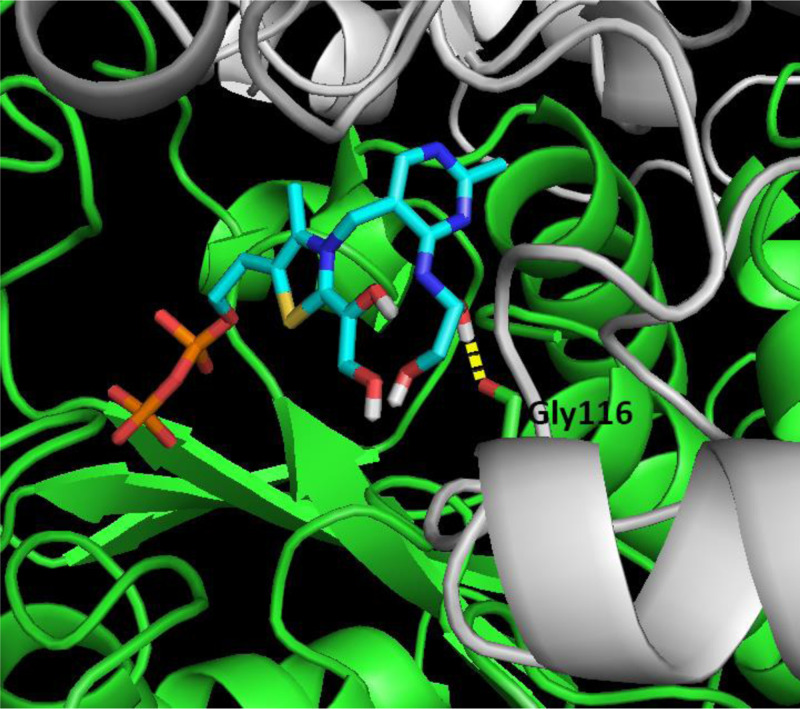
Model of the TK active site with ThDP-GliA intermediate Two GliA residues are covalently bound to ThDP via the amino group of the aminopyrimidine ring and the C2 of the thiazole ring. ThDP and GliA atoms are colored as follows: “C” - blue, N” - dark blue, “P” - orange, “S” - yellow, “O” - red, “H” - white. The enzyme subunits are shown in green. Most of hydrogen atoms of the cofactor are hidden for clarity.

With the aid of 3D computer modeling we have shown that the holoTK active site can easily accommodate two GliA residues, one at the amino group of the pyrimidine ring and the other, at the C2 site of the thiazole ring of ThDP (Figure 4). The preliminary model of an active site with two GliA residues had small overlap of atomic radii of carboxylic oxygen of glycine-116 and one hydroxyl oxygen of GliA. But molecular dynamics removed thе overlap showing that loop Gly116-Pro117-Leu118 is quite flexible.

**Scheme 5:**
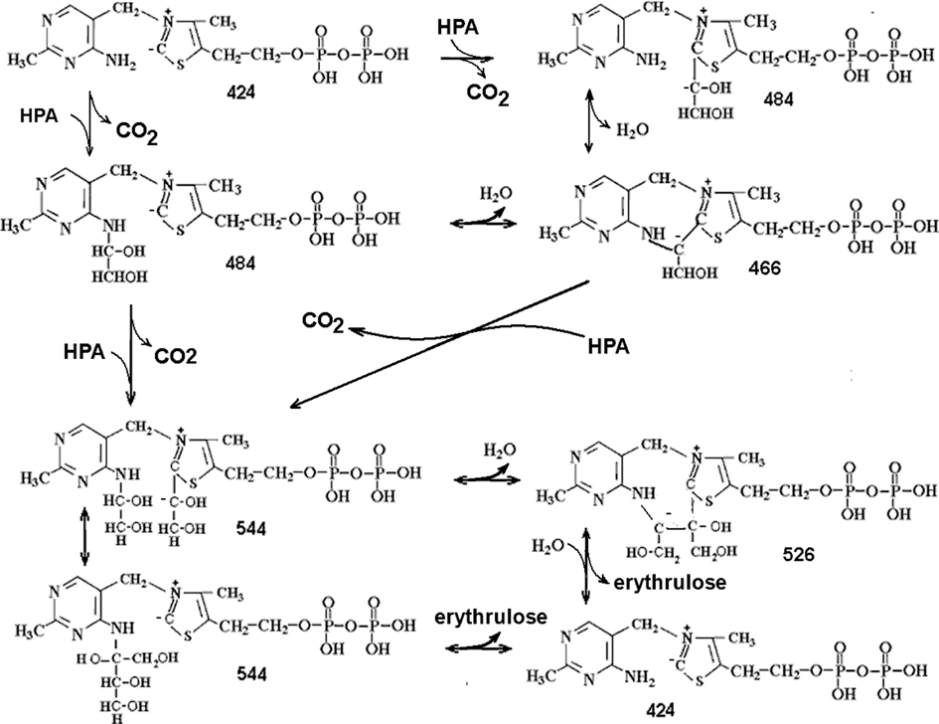
The proposed scheme of the one-substrate transketolase reaction The masses are given for non-charged molecules, differing by 1Da from masses discovered in this study.

The 3D modeling has also shown that, the GliA on thiazole would block access from the outside to the amino group thus ruling out option 2 of the initial hypothesis, namely that the second GliA binds on the aminogroup after the first one is bound to thiazole (see ‘Introduction’ section). Therefore, in view of the presence of the tricycle and the equilibrium between the alternative binding sites for GliA at the different ThDP rings, we conclude that catalysis should require a transfer of the first GliA from thiazole ring and/or its binding to the aminopyrimidine ring prior to the binding of the second substrate molecule on thiazole (Scheme 5). This is option 3 of our initial hypothesis (see introduction).

We had difficulties identifying the four-carbon derivative of ThDP: likely, erythrulose releases to the medium too fast. Another explanation is that ThDP-tetroses appear on our masspectra as masses 543.055 and 527.060 Da (Figure 1S and Scheme 1S of Supplementary Material) instead of the expected masses 543.072 and 527.077 (=425.044 + 120.0422 - 2 × 1.0077 or -18,01) due to a mass defect. Unstable ions are known to show mass defects [22,23].

In the research presented here we conclude, that HPA, in the same way as shown earlier for xylulose-5-phosphate, serves as a substrate for the one-substrate transketolase reaction, where the erythrulose product condenses from two glycolaldehyde residues formed via the decarboxylation of two HPA molecules in the absence of acceptor substrate. At least three independently obtained fragments (164.082, 180.076, and 182.092) of different forms of the main 485-intermediate prove the function of the ThDP amino group as the covalent binding site for the transferred moiety of the substrate. This function of ThDP amino group has never been demonstrated before.

## Supplementary Material

Supplementary Material

## Abbreviations

GliA: glycolaldehyde
HPA: hydroxypyruvate
TCA: trichloroacetic acid
ThDP: thiamine diphosphate
TK: transketolase
