# Supplementary material for: The mechanism of a one-substrate transketolase reaction
Source: Biosci Rep. 2020 Aug 4;40(8):BSR20180246. doi: 10.1042/BSR20180246 (PMC7403953; doi:10.1042/BSR20180246)

Appendix. Supplementary material

Additional Supporting Information may be found online in the supporting information

Supplementary information for

The mechanism of a one-substrate transketolase reaction

Olga N. Solovjeva[a], Marina V. Kovina[b], Maria G. Zavialova[c], Victor G. Zgoda[c], Dmitrii S. Shcherbinin[c], German A. Kochetov[a*]
[a] Belozersky Institute of Physico-Chemical Biology, Lomonosov Moscow State University, 119992 Moscow, Russian Federation,
[b] Sechenov First Moscow State Medical University, Ministry of Health of the Russian Federation, Trubetskaya Street 8/2, 119991 Moscow, Russian Federation,
[c] Institute of Biomedical Chemistry of Russian Academy of Medical Sciences, Pogodinskaya 10, 119121 Moscow, Russian Federation
*Correspondence and requests for materials should be addressed to G.A.K. (email: kochetov@belozersky.msu.ru)

Fig.1SA shows the wide range scale (480-550Da) of a typical ESI-MS spectrum of ThDP and its intermediates which are formed in the reaction of holoTK with HPA in the absence of $NaCNBH_3$. To make this spectrum more readable we deleted minor peaks with amplitude below 20% of the 485.065 peak amplitude, even considering the risk to lose some important peaks which sometimes go as minor (for example, the peaks 483.050 and 467.056). However, despite this sacrifice, there are still enormous amounts of foreign peaks here because of the high content of polluting traces, coming from reagents, decaying protein, column and products of their interaction. Mass spectra in the presence of $NaCNBH_3$ are similarly unreadable (not shown). Without refinement of these spectra we could only investigate known and expected peaks 483.050, 485.065, 487.081, which were reproducibly found indeed in most of initial experimental spectra after extending the scale (Fig. 1 of main text).

However none of multiple spectra had expected masses of tetrose derivatives of ThDP (545.087, 543.072 and 527.077). We had to clarify, whether they just disappeared or were converted into compounds with different masses. To answer this question we excluded foreign and insignificant peaks via the following refinement procedure. First, we deleted from the mass spectrum given on Fig. 1SA all masses which were also present in mass spectra of buffer and holoTK control. However, the resulted differential spectrum still had too many peaks (Fig.1SB), most of which we could not identify. On the next step of refinement we used the criteria of reproducibility: the essential peaks must be present in all experiments conducted under the same conditions while at different dates with different batches of reagents, columns, protein etc.

Fig. 1SC shows the synthetic spectrum obtained by uniting (intercrossing) two independent spectra obtained from two independent experiments of the same settings (without $NaCNBH_3$) but with several months interval between them. Only peaks whose masses coincide at 3 digits after the dot are

presented in the synthetic double spectrum (Fig.1SC). As we see, the peak quantity dropped significantly. The same refinement procedure was performed for cyanborohydride positive spectra with the same excellent result (Fig.1SD). Now we had 9 refined peaks in total, and three of them (483, 485 and 487) we identified and discussed in the main text of the manuscript. From the remaining 6 peaks three, 501.060, 527.058 and 543.053, were fully reproducible both in non-reducing and reducing conditions as it is seen on tetra-spectrum, obtained via crossing two double spectra Fig.1SC +Fig.1SD. 4 (Fig.1SE).

The first of these three peaks, mass 501.0603 can be identified with high precision of 0.0005 as hydrated 483.050 mass: 483.0503+18,0105= 501,0608.

Supplementary Fig. 1S Mass spectra of intermediates formed in the one-substrate reaction.

A - Typical ESI-MS spectrum of ThDP and its intermediates formed in reaction of holoTK with HPA in the absence of $NaCNBH_3$ (experiment 1). Conditions are same as for the Fig.1 of main text. Minor masses (with amplitudes below 20% of the main 485 intermediate amplitude) are excluded.

B - Same as A, but from initial spectrum are deleted all masses which were also present in mass spectra of buffer and of holoTK control.

C – The intercrossing of two B-spectra obtained in two independent experiments (experiment 1, black lines + experiment 2, blue lines) of the same setting without $NaCNBH_3$. Only peaks whose masses coincide at 3 digits after the dot are presented in the synthetic double spectrum.

D – Same as C but in presence of $NaCNBH_3$, the intercrossing of experiments 3 (green line) and 4 (orange line).

E – Coincident peaks from all four experiments are presented by tetra-spectrum, the intercrossing of C and D. Experiments 1-4, respectively: black, blue, green and orange lines.

Fig. 1SA

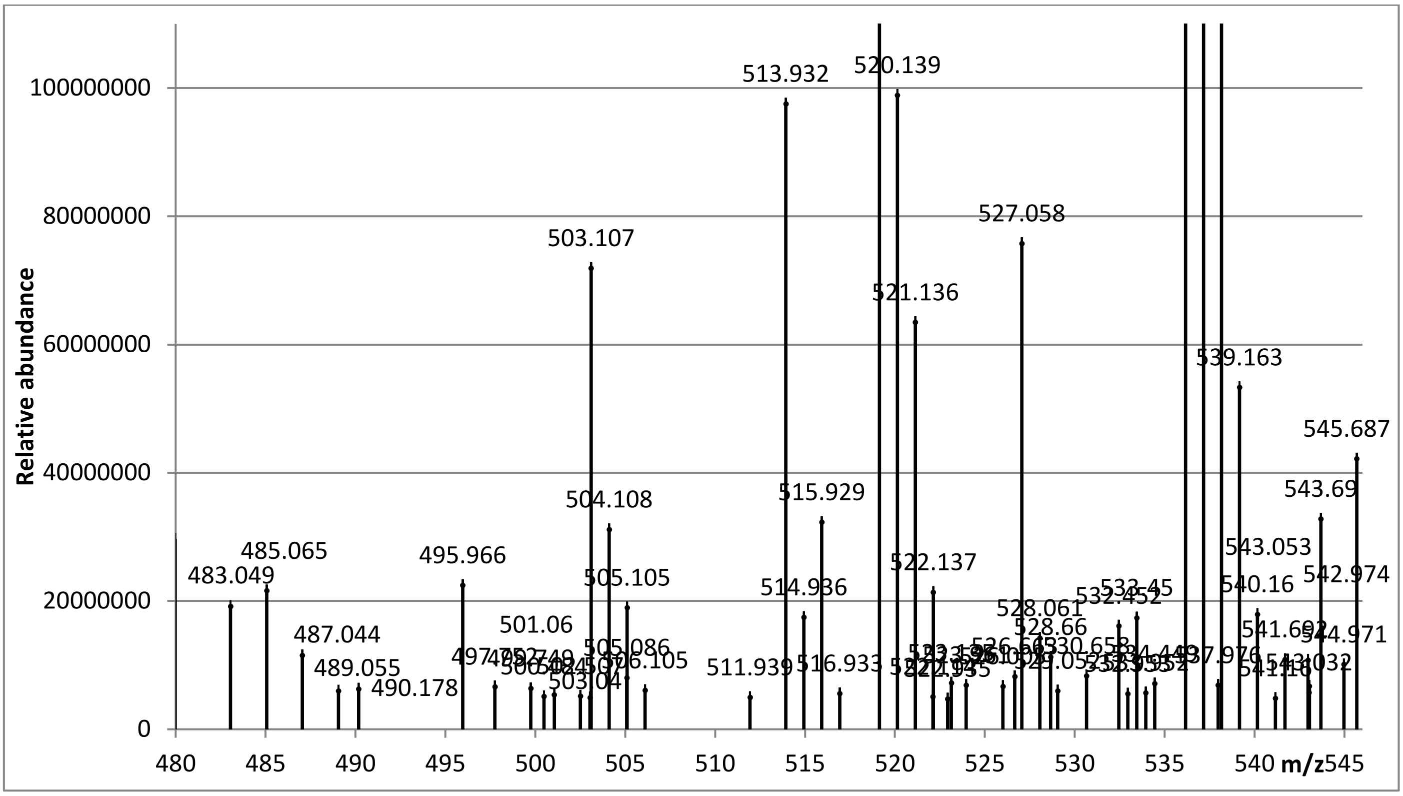

Fig. 1SB

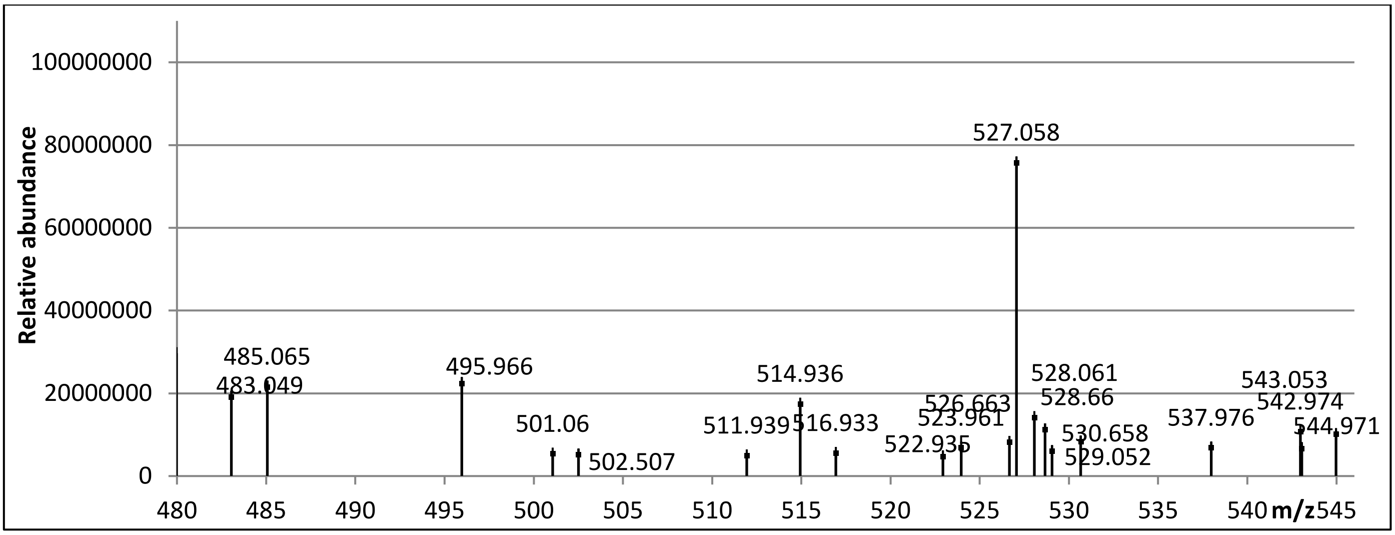

Fig. 1SC

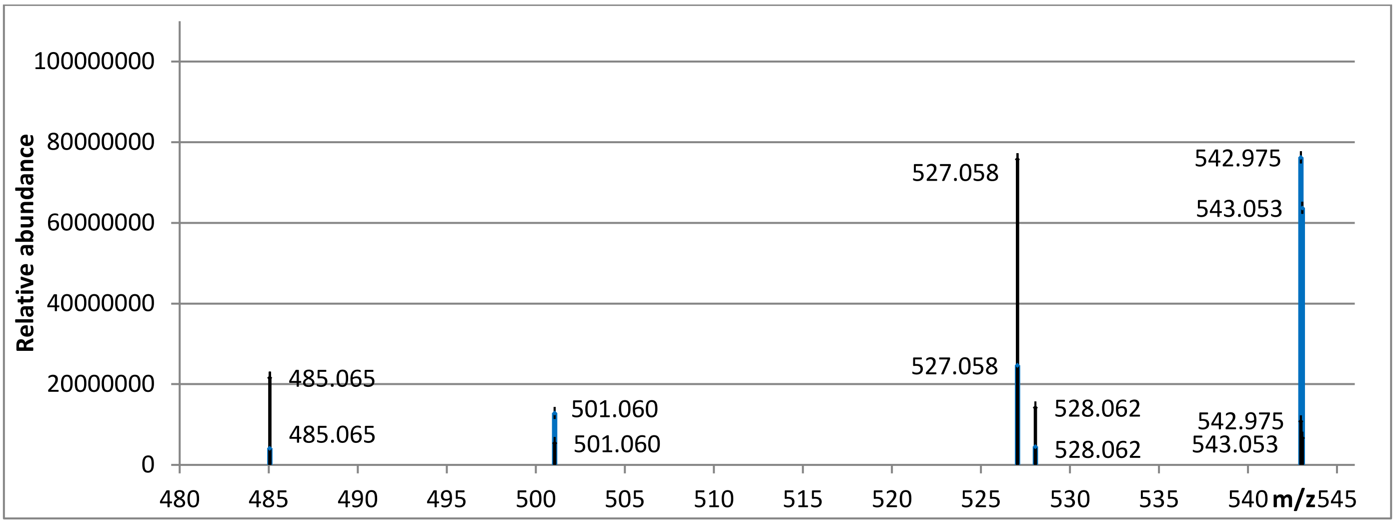

Fig. 1SD

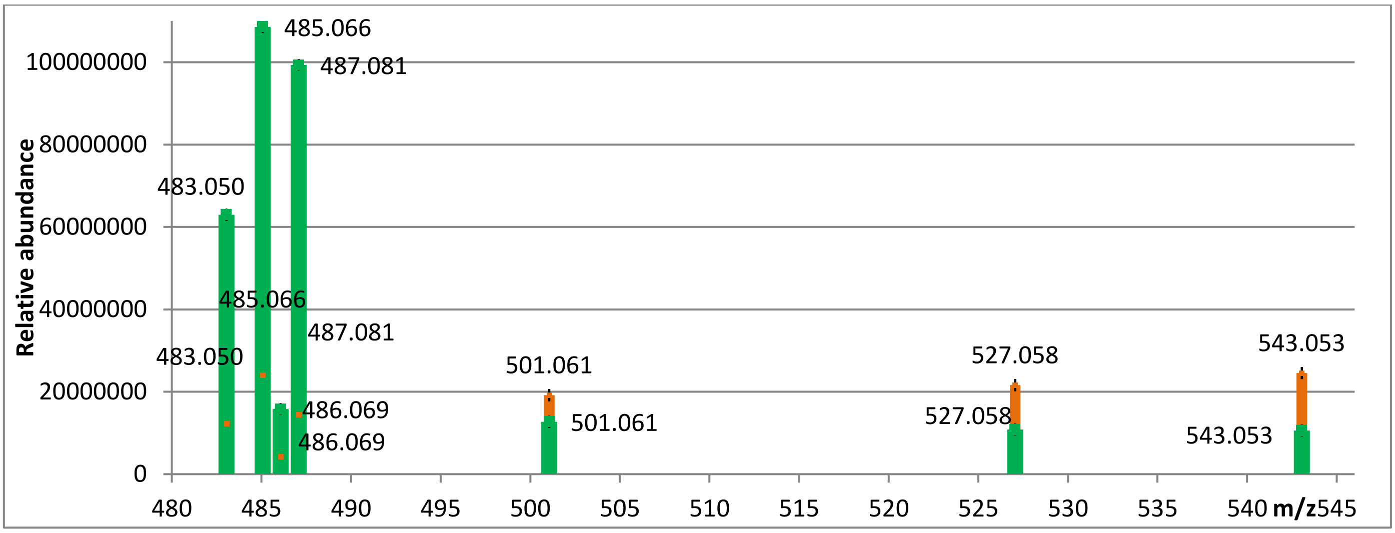

Fig. 1SE

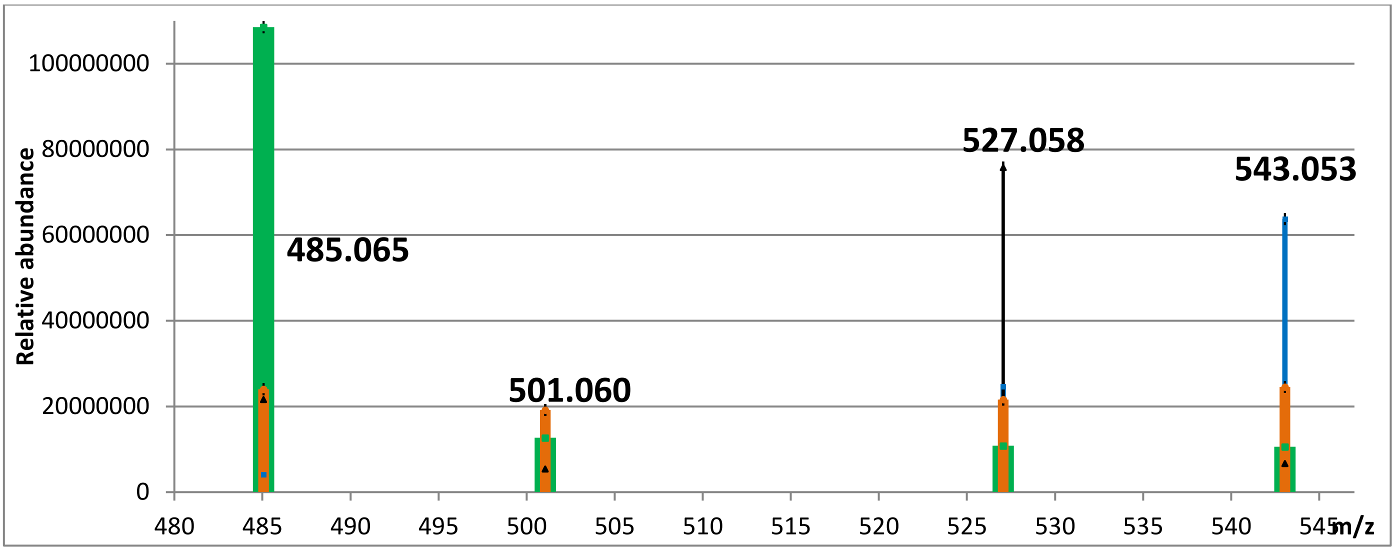

Finally, two highly reproducible major peaks 527,0583 and 543,0532 are very close to our lost ThDP-tetrose mass 545.087 without 2H or water:

545.087 – 2 x 1.0078 – 15.995 = 527.077

545.087 - 2 x 1.0078 = 543.072.

Dehydrogenation and dehydration are also shown for the main 485 peak (masses 467.055 and 483.050, respectively), and, like them, new experimental peaks 527.058 and 543.053 also differ among themselves precisely by the mass of one oxygen atom:

483.050 - 467.055 = 543.053 - 527.058 =15.995.

While fluctuations of peaks 483.050 and 467.055 between different experiments were within ±0.0005Da, masses 543.053-543.055 and 527.058-527.060 were more flexible and varied ± 0.001Da. Even though experimental masses 543.053 and 527.058 show 0.017-0.019 Da mass defect from calculated masses 543.072 and 527.077, there are no other candidates for ThDP-tetrose adducts in the mass spectra and there is no other explanation for the peaks 527,0583 and 543,0532. Therefore we fragmented these two peaks to test the guess (Fig. 2S, Scheme 1S).

The fragmentation spectra given on Fig. 2S contain precise ThDP peaks 425 and 122, thus confirming that the enigmatic 543 and 527 masses are not contaminants, they contain thiamin. The rest of detected masses are substrate-containing and show the reproducible systemic mass defect of 0.017 - 0.018 Da (Scheme 1S). In spite of the mass defect all masses fit well into the fragmentation sequence (Scheme 1S) similar to those given in the main text of the article for masses 485, 467, 425 (Schemes 2 and 3). Mass defect phenomena are known for unstable ions [22, 23]. However it still leaves a place for further investigation of the erythrulose adducts with ThDP.

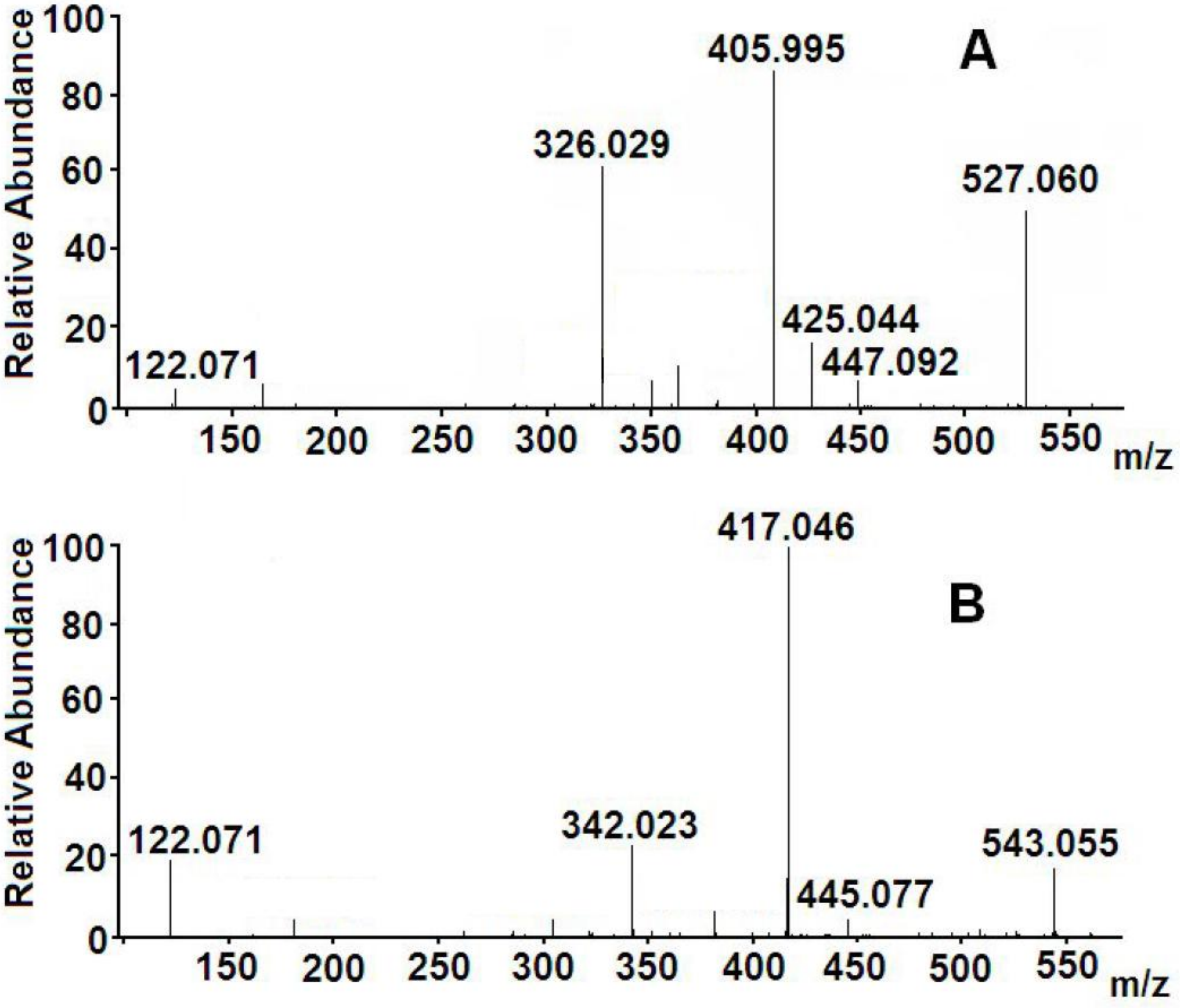

Supplementary Fig. 2S ESI-MS/MS spectrum of masses 527.060 (A) and 543.055 (B). Steadily reproduced peaks are labeled with mass value.

Supplementary Scheme 1S. Proposed structures of ThDP-tetrose family of intermediates and their fragments. The experimental masses with mass defect [-0.018] are circled and given above precise (expected) masses.

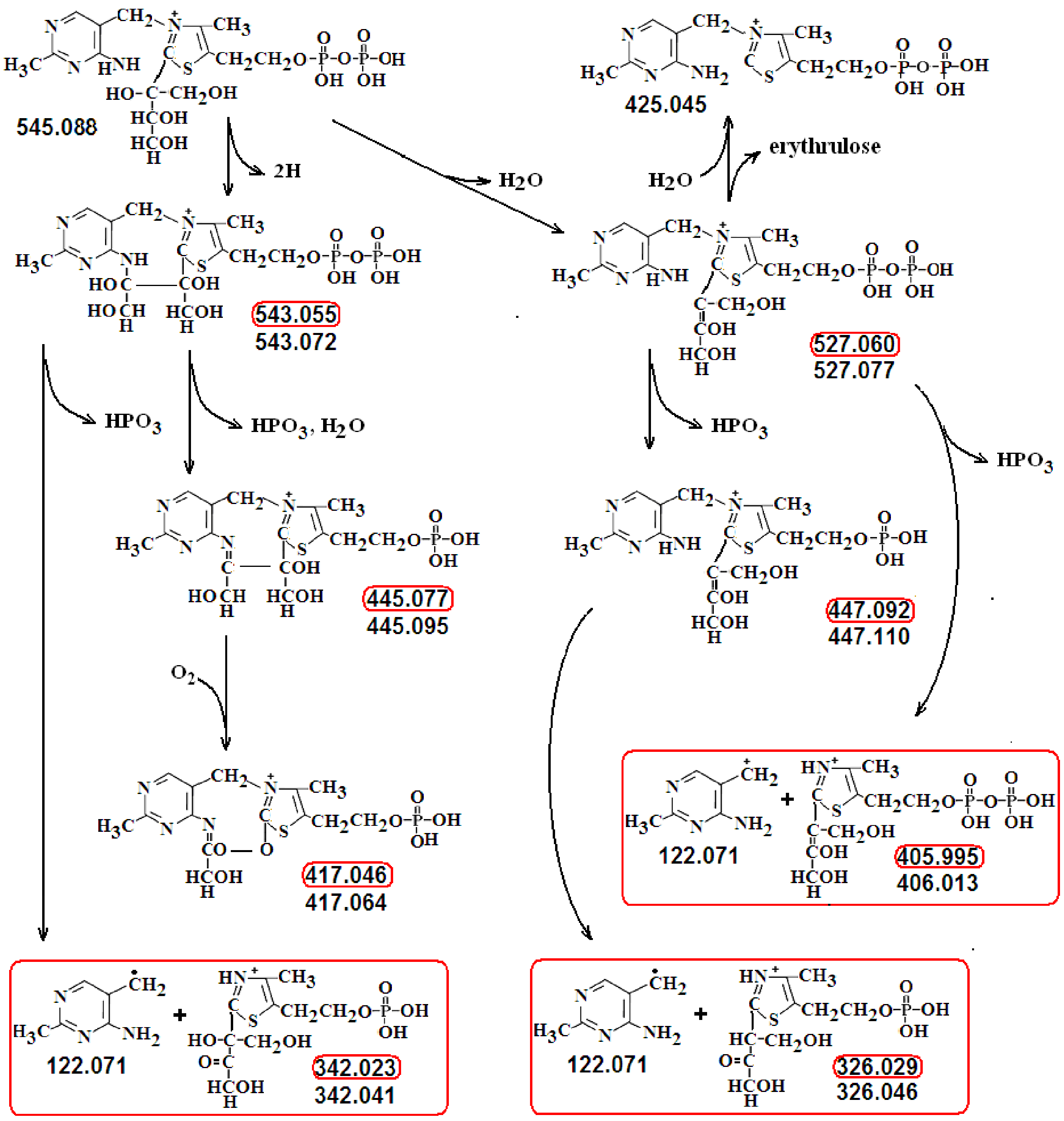

Supplement: Supplementary Material [file BSR-2018-0246-T_supp.pdf]
